# The Disappearing Lake: A Historical Analysis of Drought and the Salton Sea in the Context of the GeoHealth Framework

**DOI:** 10.1029/2020GH000271

**Published:** 2020-09-23

**Authors:** Aubrey L. Doede, Pamela B. DeGuzman

**Affiliations:** ^1^ School of Nursing University of Virginia Charlottesville VA USA

## Abstract

The Imperial Valley region of Southeastern California has become one of the most productive agricultural regions in the state and has the highest rates of childhood asthma in California. Lack of precipitation in the Imperial Valley has caused the water level of the Salton Sea to recede to a record low since its formation in the early 1900s. Previous studies of wind and dust deposition conducted in other regions have shown how reduced precipitation, ground heating, and the diminishing water level in an arid climate pose a risk of exposing previously sequestered toxic chemicals to open air, adversely affecting lung health. The purpose of this study is to draw historical parallels between the Aral Sea and Salton Sea in the context of geomorphology, ecology, human health, economics, and human migration, to inform an assessment of environmentally related health impacts of those living in the Imperial Valley region. Future droughts and heatwaves are expected to rise in frequency and severity, disproportionately affecting those impacted by financial and health disparities. Future research must include the implications of population health in the context of GeoHealth as a result of the most recent drought and the receding water levels of the Salton Sea.

## Key Points

- Comparisons between the Imperial Valley in Southern California and the Aral Sea environmental disaster in central Asia may be drawn to identify the potential human health hazards
- Financial and health disparities among local residents who live near the Salton Sea may be exacerbated due to the exposure to airborne toxins
- A GeoHealth lens of analysis is warranted in the evaluation of human health and sustainable resource allocation measures within the Imperial Valley region

## 1 Introduction

**Figure 1:**
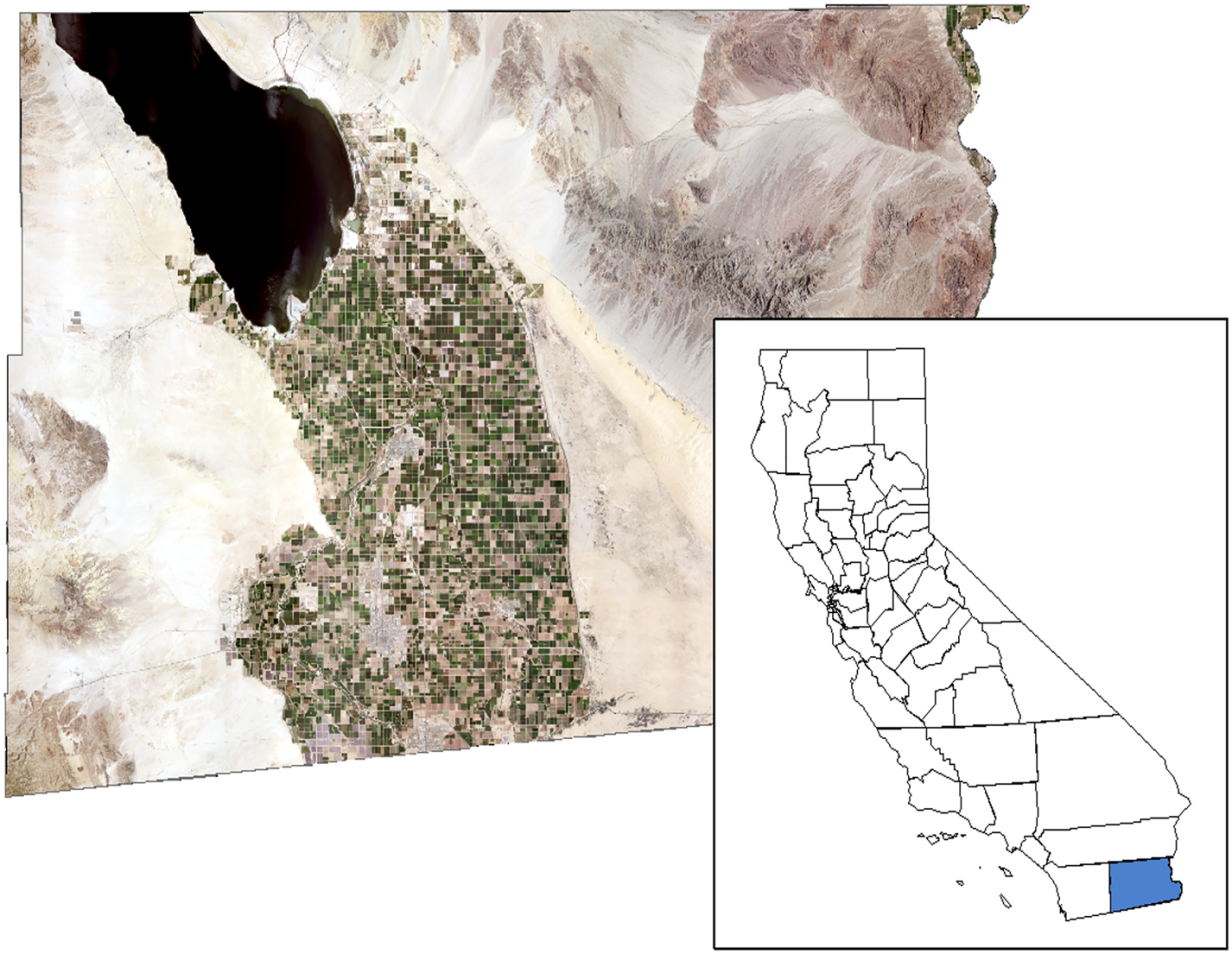
Satellite imagery of the Imperial Valley within California's Imperial County and nearby Salton Sea.

In 2011 and 2012, while the California drought was approaching peak severity, Imperial County's rate of asthma‐related emergency department admissions for children was among the highest in California and double that of the entire state (Arballo et al., 2014), putting nearly 52,000 children in the area at risk of health consequences from dry and dusty air (Bureau, U. S. C, 2015). Human activity and the construction of elaborate infrastructure for irrigation has led to the Imperial Valley region (contained within Southeastern California's Imperial County) to become one of the most productive agricultural regions in California despite its naturally arid climate. The water level of the Salton Sea, located centrally within Imperial County (Figure 1), has been diminishing as a result of evaporation and decreased precipitation and river flow. California's most severe drought took place between the years 2012 and 2017 (Barreau et al., 2017; Griffin & Anchukaitis, 2014), including four of those years under a government‐declared state of emergency.

Contamination of the Salton Sea with fertilizer and pesticide compounds from agriculture have the potential to contribute to worsened asthma symptoms (Bloudoff‐Indelicato, 2012) and may already be placing children at risk for diminished lung health. In an arid climate, a reduction in precipitation and associated ground heating (resulting from the diminishing water level and exposed lake bed) can increase airborne particulate matter, which is known to adversely affect lung health (Christian‐Smith et al., 2015; D'Amato & Cecchi, 2008). Airborne pollutants impact the long‐term lung health of children and adolescents who live in areas with high pollution rates and are more susceptible due to increased time spent outdoors (Gauderman et al., 2002, 2004). Today, Imperial County has the highest rates of asthma in children compared with the entire state of California. Future droughts and heatwaves are expected to rise in frequency and severity. Climate events often disproportionately affect those already impacted by financial and health disparities and may be more severe for individuals living in lower‐income communities, such as in Imperial County, with fewer resources to avoid or respond to environmental changes (Cook et al., 2014; Costello et al., 2009; Griffin & Anchukaitis, 2014; O'Connor et al., 2014).

Health‐related consequences of the California drought in this region have gained attention in recent years (Johnston et al., 2019); however, the specific environmental changes related to drought and their effects on human health remain largely understudied. Future research in this area may be guided by comparing the Salton Sea with similar, highly studied regions. A number of notable consequences from droughts and resource mismanagement worldwide have contributed to detrimental health effects in humans (Gomez et al., 1992; Hefflin et al., 1991; Kelley et al., 2015; Smith et al., 2014). Notably, regional characteristics, desiccation, and drought‐related health impacts of the Aral Sea Basin in central Asia have been well documented and offer multiple opportunities for comparisons to the Salton Sea and the health risks that are placed upon residents of the Imperial Valley. In this paper, the phenomenon of the Aral Sea, including its geomorphological properties, economic history, political context, and pathways of human exposure to toxic contaminants as a result of drought and water overallocation is explored in the context of its similarities to the Salton Sea's current state of desiccation and decline. The purpose of this study is to draw historical parallels between the Aral Sea and Salton Sea to inform an assessment of potential environmentally related health impacts of those living in the Imperial Valley region.

### 1.1 The Planetary Health/GeoHealth Framework

The GeoHealth framework provides an ideal context for the current research. The emerging field is based on the assumption that human, animal, and ecosystem health should be addressed jointly in order to address the root causes of environmental decline and human disease (Frumkin, 2017). This emerging framework suggests that human health is affected by a set of environmental conditions and that the disruption of natural and ecological systems by human activity are the drivers of environmental changes, including pollution, biodiversity loss, land degradation, resource scarcity, and climate change. These drivers interact in complex ways, both with each other and with proximate causes of human health effects, including exposure to natural hazards (Myers, 2017). The health and integrity of the Salton Sea's local ecosystem should be considered within the context of the health of the population living near the Salton Sea, and likewise, the population's health should be considered within the context of possible environmental conditions and exposures.

## 2 Historical Context: The Aral Sea

The disappearance of the Aral Sea during the 1960s and1970s has been referred to as a “quiet Chernobyl” (Glantz & Figueroa, 1997) and has been referred to as one of the worst environmental events of the past century (Program, U. N. E, 1992). Accelerated by unsustainable irrigation and water management practices during the mid‐1900s, the Aral Sea underwent a desiccation process that resulted in a statistically significant increase in respiratory disease in countries surrounding the Aral Sea and beyond (O'Hara et al., 2000; Wiggs et al., 2003). In addition, residents in the region experienced higher rates of cancer, hepatic and renal diseases, and pregnancy complications than ever before, resulting from toxic chemicals in drinking water from farm runoff (Whish‐Wilson, 2002).

### 2.1 Geology, Water Allocation, and Agricultural Economy of the Aral Sea Basin

The Aral Sea is an endorheic lake in central Asia, crossing the current country borders of Kazakhstan and Uzbekistan. It is primarily supplied by two major rivers, the Syr Darya and Amu Darya, for the supply of water in the setting of an arid continental climate (White, 2013). The early 1900s marked a spike in productivity for the region, supporting large fishing and cotton industries that were a major supply of food and exports for Russia (White, 2013). This quickly necessitated the diversion of water from the Syr Darya and Amu Darya rivers toward irrigation for agriculture. Following the Second World War, the Aral Sea basin underwent further development. Referred to as “the Stalin plan for remodeling nature” (Grigoryev, 1952, p. 170), the Soviet Union was driven by the desire for self‐reliance in the production of all crops necessary to support its population (Whish‐Wilson, 2002). The plan centered around an increase of the production of water‐intensive crops in the area, particularly cotton, and, to a lesser extent, wheat and maize.

The agricultural developments and production of the twentieth century were bolstered by way of manmade canals to divert water from the Aral Sea's major feeding rivers to irrigation ditches (Aus Der Beek et al., 2011; Indoitu et al., 2015; Lee & Jung, 2018; Shukla Mcdermid & Winter, 2017; White, 2013). Priority for water resource allocation was given almost entirely to the production of crops, including the emptying of nearby reservoirs to cover any deficit (O'Hara, 2000). This effectively ended the nomadic tradition of Indigenous populations in favor of settling and cultivating farmland in the setting of increased immigration of farmers to the area (White, 2013).

### 2.2 Aral Sea Desertification

The Cold War era intensified the Soviet Union's need to increase irrigation and cultivation of the land independently, and by the 1980s, the Soviet Union was the second‐largest exporter of cotton in the world (White, 2013). An increased demand for irrigation to support the cotton economy continued to divert river water from the Aral Sea, and by the 1980s, no river water reached the Aral Sea during average or dry years (Whish‐Wilson, 2002). Between 1960 and 1989, the Aral Sea's water volume diminished by over half (White, 2013).

**Figure 2:**
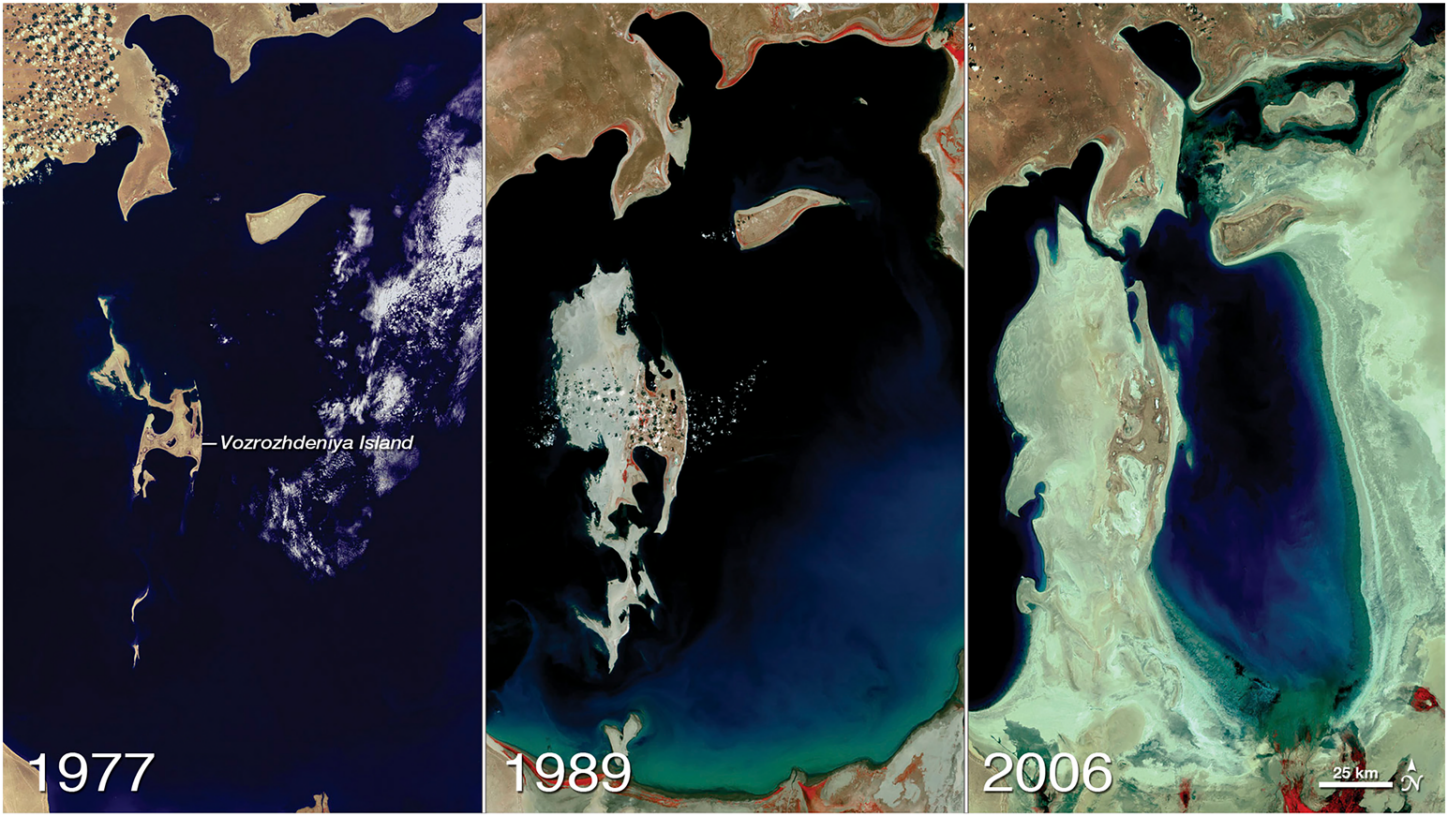
Landsat satellite images showing the gradual desiccation of the Aral Sea. Source: NASA Earth Observatory (Observatory, N. E, 2012).

Soviet‐era irrigation practices, combined with high evaporation during the summer months, have left behind a nearly dry and empty lake basin (Figure 2) (Indoitu et al., 2015). Today, the Aral Sea totals less than half of its surface area and a quarter of its volume since the 1960s (Lee & Jung, 2018), and the salinity of the remaining water has reached levels similar to ocean water (Crighton et al., 2003). The resulting Aralkum Desert has seen significant increases in extreme air temperatures and overall summer air temperatures (Indoitu et al., 2015; Shukla Mcdermid & Winter, 2017). Aus Der Beek et al. (2011) have concluded that while global climate change alone has been a factor in the desertification of the Aral Sea Basin, direct interference in the form of abstractions from the water supply have contributed to approximately 86% of the Aral Sea's dramatic reduction in its water level.

### 2.3 Human Health and Economic Impacts of the Aral Sea Desiccation

As soon as the early 1900s, the ecological ramifications of increased irrigation and agricultural activity on human health began to emerge. In addition to salinization of soils from the river water, massive diversions of water to farmland areas saturated the water table and created increased areas of swampland, contributing to malarial outbreaks in the region (White, 2013).

In addition to the desertification of the Aral Sea basin, the overambitious agricultural development on the part of the Soviet Union included the use of toxic pesticides, fertilizers, herbicides, salts, and other chemicals, such as the organophosphate phosalone and organochlorines PCB, Toxaphene, Lindane (HCH), and DDT (Crighton et al., 2003) in amounts far higher than were used elsewhere in the Soviet Union (White, 2013). Most notably, the chemical TCDD (an active ingredient in Agent Orange and a known human carcinogen) was deployed in cotton fields (White, 2013). These chemicals made their way to the Aral Sea and were confined underwater, later to be exposed as the water level diminished (Indoitu et al., 2015).

**Figure 3:**
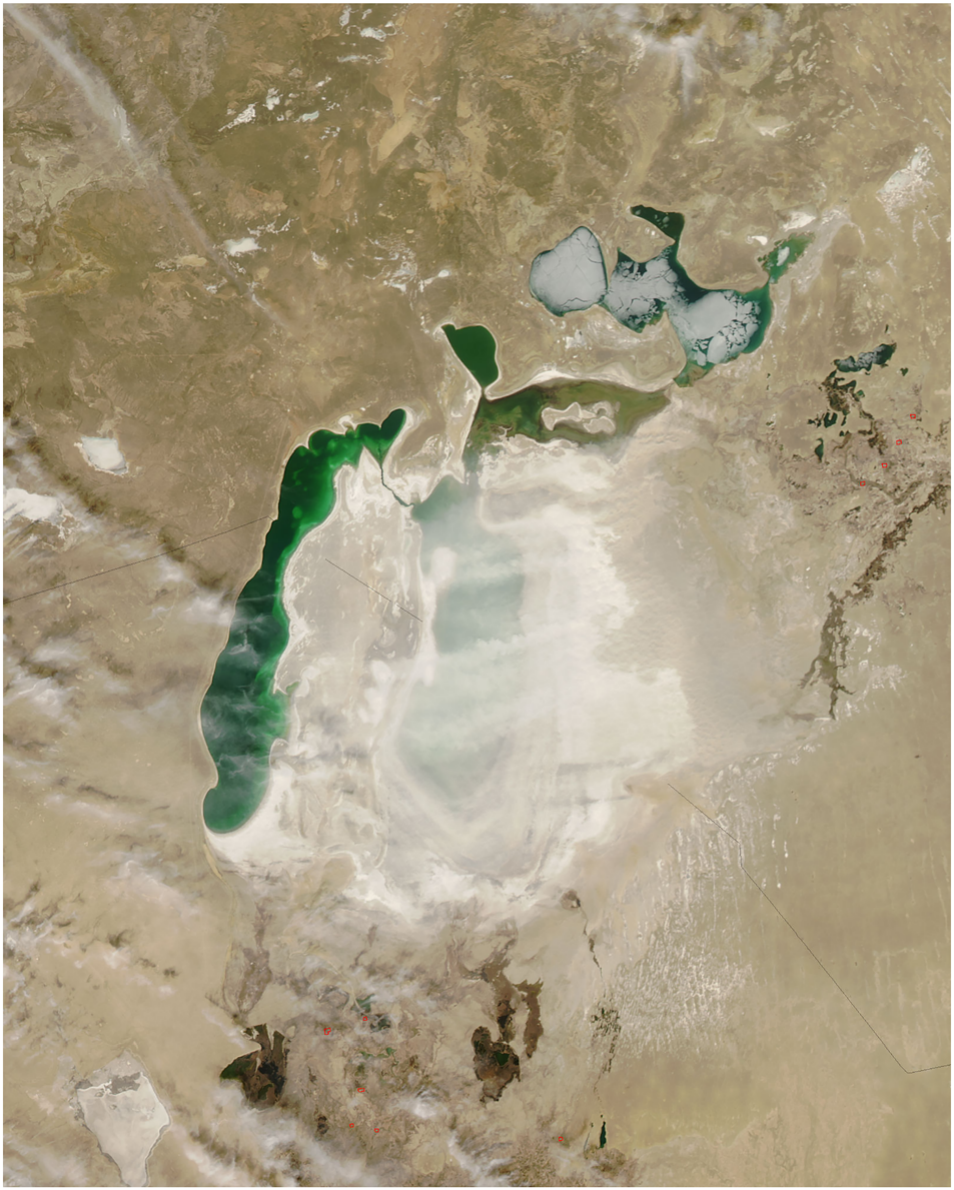
“White dust storm” over the Aral Sea. Source: NASA Earth Observatory. (Observatory, N. E, 2007).

The dry lake basin in present day can distribute these chemicals across distances reaching up to 500 km (Indoitu et al., 2015) in what has come to be known as “white dust storms: clouds of toxic dust produced when combined with dry weather and strong winds” (Figure 3). White dust storms have been increasing in frequency and severity in the Aral Sea Basin (Indoitu et al., 2015; Shukla Mcdermid & Winter, 2017) at the same time that they have become less frequent in the general area (Indoitu et al., 2015). As a result, the rate of dust deposition, containing high concentrations of toxic chemicals, is among the highest in the world (O'Hara et al., 2000) and has infiltrated not only the air but also the water and food supply pathways (Crighton et al., 2003; Kaneko et al., 2003).

Recent evidence (outlined in Table 1) demonstrates the multiple human health and environmental effects that have arisen as a result of the Aral Sea's desiccation process. Furthermore, the recession of the Aral Sea's water level between 1960 and 1970 (Crighton et al., 2003) and the resulting increase in water salinity caused the collapse of the region's fishing industry. This forced mass out‐migration from the area and lasting high rates of unemployment and economic hardship (Crighton et al., 2003). In the setting of economic collapse and health concerns, those who were able to do so migrated away from the area, leaving behind a marginalized population that did not possess the resources to relocate (White, 2013).

**Table 1:** Review of the Environmental and Human Health Impacts in the Region of the Aral Sea Basin

Authors	Study area(s)	Health assessment	Environmental assessment	Findings (health)	Findings (environmental)	Study limitations
Bennion et al. (2007)	Karakalpakstan, Uzbekistan	Questionnaire‐based assessment of household exposures and self‐reported respiratory health (asthma, allergic rhinitis, pneumonia)	Dust deposition rates (PM_10_ and PM_2.5_) within 5 km of study populations	Some evidence for an inverse relationship between FEV_1_ and dust exposure, but no significant relationship was found	Highest rates of dust deposition occurred during the summer months and in the region closest to the original shoreline	Unable to test difference between asthma and allergic rhinitis
						Cross‐sectional study does not allow for testing for children with wheezing
					No significant variation between PM_10_ and PM_2.5_ fractions	
		Pulmonary function (FEV1) collected via portable spirometer				
Crighton et al. (2003)	Karakalpakstan, Uzbekistan	Questionnaire‐based assessment of perceived environment, social support networks, psychosocial health, and self‐rated somatic health (general)	None	High rates of poor self‐rated health	None	Lower‐than‐expected ratings of poor health and environmental may be due to mass out‐migration from the region, leaving behind individuals who were less oncerned about these issues
				Respondents were more likely to have poor self‐rated health if they were concerned about environmental problems and had an intermediate or higher education level		
Indoitu et al. (2015)	Aral Sea region	None	Remote sensing observations (satellite imagery, ozone mapping spectrometry) to track frequency, size, and sources of dust storms andaerosol concentrations over the region	None	The Aral Sea dry lake bed has been a strong source of dust emissions since 2000 and has included the northern and southern desert areas as dust sources	Absence of meteorological monitoring stations on the dried Aral Sea surface
					Dust storm frequency, composition, and structure have changed as a result of the Aral Sea desiccation process	
					Dust emissions originating from the Aralkum desert are capable of traveling hundreds of kilometers	
Kaneko et al. (2003)	Kazalinsk District, Kazakhstan and control area	Questionnaire‐based assessment of overall health, gastrointestinal symptoms	None	Significantly higher prevalence of gastrointestinal symptoms, abnormal renal labs in children living in the Aral Sea region vs. control	None	Unknown specific cause for renal tubular dysfunction found in the study area
		Renal tubular cell injury as measured by urine sampling				
Kunii et al. (2010)	Kazalinsk District, Kazakhstan and control area	Questionnaire‐based assessment of household exposures and respiratory symptoms (pneumonia, chest infection, wheeze)	None	Significantly higher prevalence of wheeze and restrictive pulmonary dysfunction among subjects in the Aral Sea region compared to those living farther away	None	Confounding factors related to measurement bias during questionnaire administration and weather‐related variability in pulmonary function performance
				No significant difference for asthma or obstructive pulmonary dysfunction		
O'Hara et al. (2000)	Eastern Turkmenistan	None	Airborne dust deposition rates (PM_10_) and physical/chemical composition	None	Dust deposition rates were higher in desert monitoring sites than those closer to the Aral Sea	None noted
					At sites near irrigated areas, PM_10_ values were greater	
					High levels of phosphalone (organophosphate) contamination were found across sites and were highest in irrigated areas despite the cessation of pesticide spraying	
Wiggs et al. (2003)	Karakalpakstan, Uzbekistan	Questionnaire‐based assessment of household exposures and respiratory symptoms (chronic cough, wheeze, asthma)	Dust deposition rates (PM_10_) within 5 km of study populations	Children living closer to the former shoreline had a lower prevalence of respiratory health problems compared to main agricultural and urban areas	Summer months experienced conditions (i.e., temperature, precipitation, and wind patterns) that were conducive to increased sediment erosion and dust transport, especially in the northern portion of the study area	Likelihood that dust is not the only environmental factor that may cause changes in human health
						Dust deposition data indicates multiple potential sources of dust
		Pulmonary function (FEV_1_) collected via electronic volume‐flow spirometer				
						Monthly aggregate data may mask short‐term effects on health caused by single dust events
					During the dusty season, deposition rates of PM_10_ far exceeded US EPA standards	

Of all the regions making up the former Soviet Union, the Aral Sea region has been recorded as having the highest rates of tuberculosis, far exceeding the classifications of an epidemic outbreak (Wiggs et al., 2003). Based on evidence of dust deposition patterns as demonstrated by Bennion et al. (2007) and Wiggs et al. (2003) and surveys of respiratory symptoms and illness, it is possible that dust in the Aral Sea region was the cause of these symptoms. Infant mortality rates in the region are among the world's highest, exceeding 100 deaths per 1,000 live births (Wiggs et al., 2003). In children, autopsy results have shown a strong relationship between proximity to the Aral Sea region and lung tissue changes, including fibrosing alveolitis and damage of interstitial lung tissue (Kunii et al., 2010).

## 3 California's Imperial Valley and the Salton Sea Basin

In the region of the Aral Sea basin, the economic, environmental, and human‐health impacts on the local population could be seen as a warning for other areas of the globe facing similar processes and impacts. Though not connected by people or place, the Aral Sea and Salton Sea regions are comparable through similar geologies, economic goals, strains on the natural systems, and impacts of the local and regional environments on human health.

### 3.1 The Salton Sea: Geology, Water Allocation, and Agricultural Economy

The Salton Sea in California's Imperial County, like the Aral Sea, is an endorheic geologic depression located at the northern end of the Imperial Valley and lies over 200 feet below sea level (Cohn, 2000). The Salton Sea was preceded by the much‐larger ancient water body, Lake Cahuilla, which underwent repeated expansion and contraction due to repeated flooding from the Colorado River during prehistoric times and ultimately dried up completely by the sixteenth century (Cohn, 2000; Laylander, 1995). The resulting Salton Sea basin (also known as the Salton Depression) remained dry until 1905, when a faulty canal gate, meant to corral the Colorado River for irrigation, flooded the area and resulted in what is currently the Salton Sea (Cohn, 2000; Xu et al., 2016). At 35 miles long, up to 15 miles wide and holding approximately 7.5 million acre‐feet of water, it is currently the third‐largest saltwater lake in North America (Cohn, 2000).

As with the Aral Sea's two main feeding rivers, the Imperial Valley's Colorado River serves as the main water source for large human developments in an arid climate and is bolstered by a complex system of canals. Seven states in the southwestern United States—five of which are some of the fastest growing states in the country—depend on the Colorado River, whose major landmarks include Lake Mead and the Hoover Dam (which supplies the Las Vegas area) in addition to the Salton Sea. Though the Salton Sea's heyday was marked by beachfront properties and images of the Hollywood elite visiting the “miracle in the desert” on holiday, this massive body of water has no natural feeding rivers to maintain its water level due to the accidental nature of its modern existence (Goodyear, 2015; Xu et al., 2016).

In the midst of its time as the center of a booming tourist destination, the original purpose of the Colorado River canal system also gave way to a flourishing agricultural industry, thanks to the seemingly endless supply of water from the river. Use of water from the Colorado River, as with the Amu Darya and Syr Darya of the Aral Sea Basin, was propagated by the United States government, incentivized by the prospect of irrigation and agricultural production in an area offering a year‐round growing season (Arballo et al., 2014).

The mechanisms of water inefficiency in agriculture between the Aral and Salton Seas differ politically. One was a result of the Soviet Party's economic and political need for increased production. Meanwhile, the Salton Sea's existence has been maintained by a political chokehold held by local farming lobbies on the government's sustainability efforts, making it virtually impossible for water usage regulations to be enforced. The result in both areas has been an unsustainable use of water resources toward the production of water‐inefficient crops in the context of the local climates due to the lack of incentives for conservation.

The Colorado River was first turned into a source for California agriculture during a particularly wet year, setting off a history of excessive and unsustainable water use (The Economist, 2014). The interpretation of water from the Colorado River by farmers as a birthright to farmers was carried forth by the Law of the River agreement in 1922, which established California's share of the River's water (shared by Colorado, Nevada, and Utah) and set the price of its agriculture‐designated water at $0.20 per gallon (Goodyear, 2015). In Imperial Valley in 1924, the Salton Sea was designated as an agricultural sump as a result of irrigation runoff from nearby farmland. Despite the naturally dry climate and relative lack of a natural local water supply, the low price of water—left unchanged to this day—did little to discourage the production of water‐intensive crops and use of flood irrigation by farmers in the area (Goodyear, 2015).

The once‐thriving beachfront communities along the Salton Sea, dependent on the ebbs and flows from farmland irrigation, eventually became ghost towns, their piers for jet skis and fishing now ending hundreds of feet from the receding shoreline. Despite the fate of these vacation towns, water from the Colorado River continued to feed the vast swaths of farmland in the Imperial Valley, at one point pulling approximately 5.2 million acre‐feet of water from the Colorado River (Cohn, 2000). Today, the Imperial Valley is still one of California's most productive agricultural areas and holds the largest water right from the Colorado River (Arballo et al., 2014). As a result of the arid climate and irrigation from the 475,000 acres of farmland in Imperial Valley (Cohn, 2000; Orlando et al., 2006), 75% of water inflow to the Salton Sea now originates almost exclusively from agricultural drainage from Imperial Valley via two southern streams in Imperial County and a third originating from Riverside County, to the north of the Salton Sea (Orlando et al., 2006; Xu et al., 2016).

### 3.2 Desiccation, Ecological Impacts, and Current State of the Salton Sea

**Figure 4:**
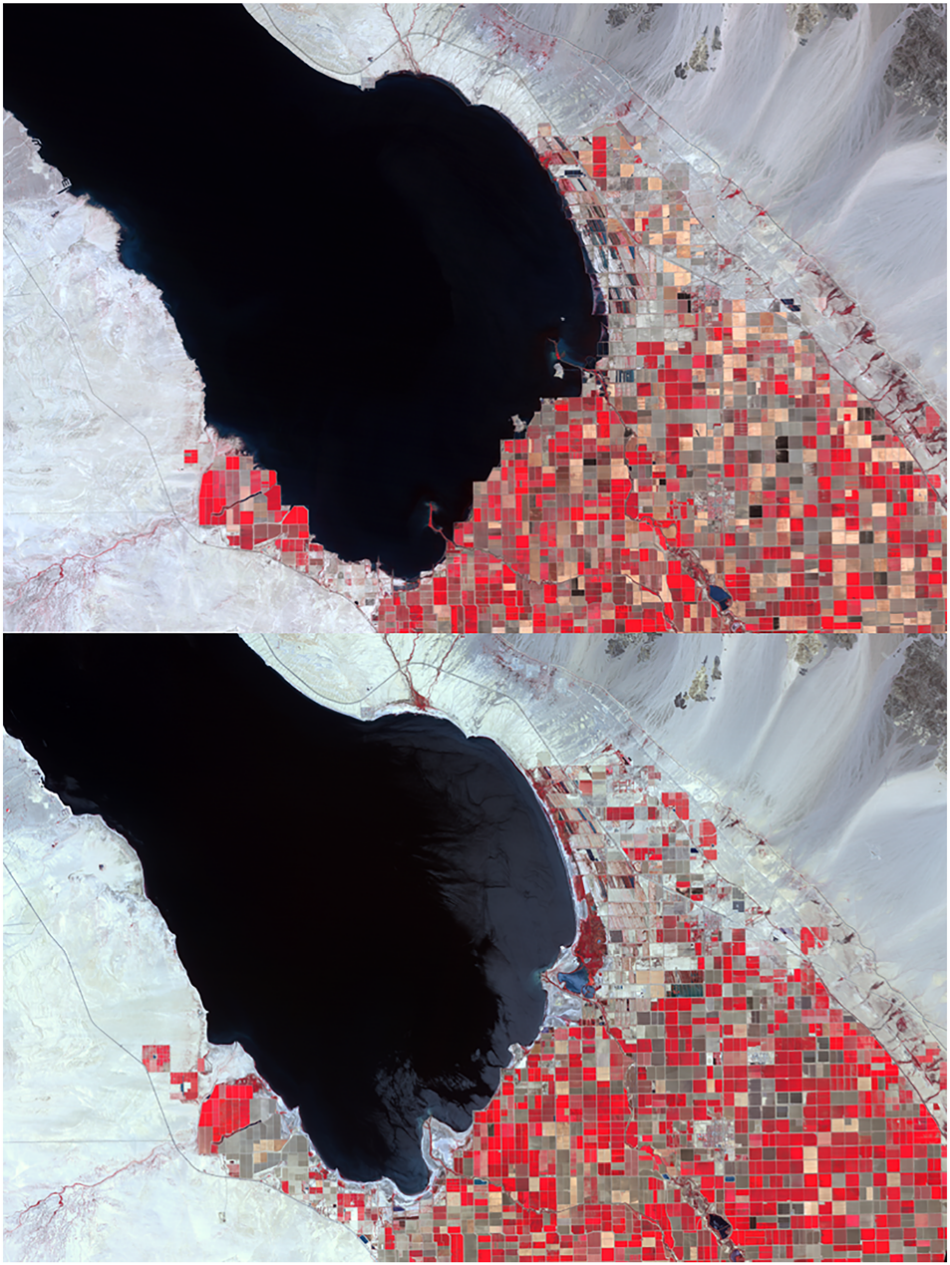
Evidence of the Salton Sea's diminishing shoreline from 1984 (top) to 2015 (bottom). Source: NASA Earth Observatory (Observatory, N. E, 2015).

As in the Aral Sea region, the lack of precipitation in the Imperial Valley region has caused the water level of the Salton Sea to recede (Figure 4). Years of recent water scarcity have forced farms to conserve of water, and in 2013, half a million acres of farmland were left fallow due to drought conditions (The Economist, 2015). This led to the reduction of irrigation runoff into the Salton Sea and is compounded by the natural evaporation from the water surface.

Though not a direct comparison to the Amu Darya and Syr Darya rivers, whose natural water supply was diverted away from the Aral Sea for irrigation nearby, the impact on the seas' salinity and ecology are comparable. The Salton Sea's water level is currently at a record low in the modern era. Since its creation, the sea has since become home to abundant populations of water fowl, including some species of endangered birds, that are dependent on this body of water along the Pacific flyway (Cohn, 2000). However, salinity of the lake, most recently measured at 45 g/L, is already 25% more saline than the Pacific Ocean (King et al., 2011). This, combined with frequent algal blooms and depletion of the water's dissolved oxygen content, has made survival a challenge for most animals that call the lake home (Cohn, 2000; Kaiser, 1999). The loss of this habitat is significant, as the loss of California's natural wetlands due to human development has made the Salton Sea a critical ecological resource (Cohn, 2000).

### 3.3 Drivers of Human Health Impacts of the Salton Sea's Desiccation Process

The existence of the Salton Sea in its current state is more than an eyesore or an ecological conundrum; the Salton Sea may also be the source of current, and likely future, health risks to the nearby human population. Similar geographic and environmental features (Gomez et al., 1992; O'Hara et al., 2000; Wiggs et al., 2003) between the Salton and Aral Seas suggest that there could be links between these features and the potential for adverse health consequences that may be applicable in the context of the Imperial Valley. Evidence of human health impacts resulting from drought conditions in the Imperial Valley of Southern California is limited to anecdotal evidence. This evidence, in the form of newspaper articles and editorials, highlights the respiratory health of residents in the area and the impact of health disparities on families' inability to effectively cope with environmental risk factors (Bloudoff‐Indelicato, 2012; Goodyear, 2015; Ketcham, 2012).

Local socioeconomic statistics suggest that residents in the Imperial Valley are susceptible to poverty‐related health risks associated with living in the region, such as asthma. The 2013 median family income of Imperial County was approximately 25% below the median national family income, and 23.3% of families were below the federal poverty level, compared to 15.9% in the United States (Arballo et al., 2014). Individuals and families with fewer economic resources may be unable to avoid exposure or seek treatment (e.g., adhering to asthma guidelines) in order to reduce exposure to poor air quality (Bureau, 2015).

Diminishing water levels in the Salton Sea poses a risk of exposing previously sequestered toxic chemicals to the open air. Both the Aral and Salton Seas are terminal lakes in dry, arid climates. The use of agricultural chemicals on nearby farmland in both regions has accumulated in each region's body of water (Table 2), and these previously submerged chemicals have been increasingly exposed to the open air due to water evaporation. Studies have found these chemicals in the air surrounding the Aral Sea. Rivers feeding the Salton Sea drain pesticides, including DDT, chlorpyrifos, dieldrin, PCBs, selenium, and toxaphene, among other toxins (de Vlaming et al., 2004; Orlando et al., 2006; Xu et al., 2016). Although the Salton Sea's desiccation process is still in its beginning stages, previous studies of wind in the Imperial Valley and dust deposition from the Salton Sea's borders (King et al., 2011) have shown that the diminishing water level poses a similar risk of exposing previously sequestered toxic chemicals to the open air as evaporation continues.

**Table 2:** Review of Environmental Testing in the Salton Sea and Imperial Valley Region

Authors	Environmental assessment	Measurements	Findings
de Vlaming et al. (2004)	Presence of insecticides in water of Salton Sea's contributing rivers (Alamo River and New River)	Toxicity testing of water using mortality rates of three aquatic species	Toxicity of water samples was due to organophosphate insecticides, chlorpyrifos, and diazinon
King et al. (2011)	Relationships between season, soil properties, and windblown dust emissions	PM_10_ dust emissions, soil sampling for moisture content, and chemistry	Some degree of seasonality in Salton Sea's dust emission potential among soft crusts, producing significant dust emissions from winter to early spring, as well as minimally fluctuating emissions from dry wash surfaces
			No correlation between PM_10_ emissions and soil composition/texture, though dry wash sites consistently produced higher PM_10_ emissions compared to other landform types
Orlando et al. (2006)	Presence of pesticides in water and suspended sediment in Salton Sea's contributing rivers	Gas chromatography/mass spectrometry for detection of organochlorine pesticides	Over 75% of samples contained chlorpyrifos, DCPA, EPTC, and trifluralin
			Samples from the Alamo River contained maximum dissolved concentrations and contained greater numbers of pesticides compared to the New River samples
			Maximum concentrations of carbofuran, chlorpyrifos, diazinon, and malathion were higher than U.S. EPA aquatic life benchmarks
Xu et al. (2016)	Presence of contaminants in Salton Sea water, its feeding rivers, sediment, and fish tissue	Toxicity testing of water	Water and sediment samples showed contamination by DDTs, PAHs, chlorpyrios, pyrethroid insecticides, copper, and chromium
			While tributary river water was more contaminated than water in the Salton Sea, the Salton Sea's sediment showed higher levels of contamination than river sediment
			Fish tissue samples showed contamination of DDTs, selenium, and chlorpyrifos

The similarities between the Aral and Salton seas are further evidence of the human effects of drought in a region that is geographically and climatologically similar to the Salton Sea. Additionally, these regions underwent or are currently experiencing very similar farming and irrigation practices. Although the parallels between specific characteristics of both populations are not exact, both regions contain or have contained populations who are socioeconomically vulnerable to climate conditions, in part due to their inability to relocate. Residents of the Imperial Valley may only have started to see health effects of drought in recent years. However, historical evidence presented here suggests that attention needs to be paid to the future public health impacts of environmental pollutants on the Imperial Valley population, and water resource allocation policy should be reconsidered as a measure to impact public health.

### 3.4 The “Costs of Inaction” in the Salton Sea Region

The Aral Sea environmental crisis and its consequences on the local economic climate as well as human health and migration have been studied extensively. In the area surrounding the Salton Sea, the full extent of its impact on human health and the environment have yet to be fully appreciated. In a 2014 report, Cohen (2014) outlined the projected “costs of inaction” from a range of issues that may provide insight into the price of the decline of the Salton Sea region (i.e., the estimated costs to the public if no large‐scale mitigation efforts are implemented). Notably, the report found that the financial cost of dust emissions on the local population's health could amount to up to $37 billion USD (2014 through 2047) and between $10 and $26 billion in nonuse value costs in terms of ecological and habitat worth (i.e., the assigned value of a resource, regardless of whether or not this resource will be specifically used).

## 4 Discussion

In an effort to increase agricultural productivity, the governments in the Aral Sea and Imperial Valley regions introduced policies that included widespread irrigation projects and other support systems for agriculture at the expense of sustainable practices. In the Aral Sea basin, productivity was driven by Soviet demands and resulted in a diversion of natural feeding rivers toward agricultural land. Farmers in the Imperial Valley responded to market demands for produce and were motivated by the low price of water to produce water‐intensive crops, thereby turning the area into one of the most productive regions in the country for agricultural products. As a result of placing unreasonable demands on the environment during nondrought periods, the misuse of water resources in the Aral Sea basin has led to a collapse of the agricultural system and the economy upon which it depended. Similarly, the high productivity of the Imperial Valley's agricultural economy, relative to the state of California, suggests the consequences of drought may impact the United States on a broader scale. California is the most populous state in the United States with the country's largest economy, accounting for 12% of the population and 13% of the nation's GDP (Young, 2016). In addition, the average socioeconomic status of many residents of the area implies that in the event of a public health crisis, additional strain may be placed on public healthcare payors, such as Medicaid and Medicare. Therefore, the combination of these social and economic factors provides substantial risk for impacting a large section of the country's healthcare and economic sectors.

Evidence of the occurrence of anthropogenic change to the environment in other areas of the world, as well as the human health and economic impacts of these changes, provides a scientific premise for the investigation of similar environmental impacts on the health of California residents. Anthropogenic change to the environment, such as what occurred in the Aral Sea, has the potential to significantly impact human health and local economies. The Imperial Valley is at risk for the same consequences, and more research is needed to help clarify what these local risks might be to better inform policy moving forward. Presently, California's Governor has lifted the state of emergency due to the recent drought, initiated in 2014 (State of California Department of Water Resources, 2017). This easement should be interpreted cautiously, and not as a prediction of unfaltering future improvement of conditions. The years of 2012–2016 marked the state and region's worst drought in over a century (Griffin & Anchukaitis, 2014). Droughts associated with anthropogenic climate change are expected to recur across North America with a shortening window of opportunity to prevent or mitigate harmful human health effects (Cook et al., 2014; Costello et al., 2009; Griffin & Anchukaitis, 2014; O'Connor et al., 2014).

## 5 Conclusion

The Salton Sea shares several major characteristics with its historical partner, the Aral Sea, and suggests that the health impacts of agricultural activity in the Salton Sea region must be studied in the context of human interference with an ecosystem and water availability as a result of unsustainable farming practices. Knowledge of these similarities provides an opportunity to act accordingly to prevent the practices near the Salton Sea from similarly impacting its populations and ecosystems.

In the present day, factors such as climate change and the subsequent increase in respiratory disease incidence and severity (Sarfaty et al., 2015) have accelerated the impacts of unsustainable resource use. Therefore, the need of further interdisciplinary research within the emerging field of GeoHealth is increasingly necessary. Such research may further elucidate the impact of the health of the Imperial Valley's ecology on the respiratory health of its residents. Since the 1970s, droughts worldwide have been longer and more severe. Future droughts and extreme heatwaves are expected to continue to rise in frequency and severity, disproportionately affecting those already impacted by health disparities (Costello et al., 2009; O'Connor et al., 2014).

New research into the impacts of poor respiratory health in drought areas will provide a perspective on underrepresented environmental challenges at the local and regional levels. The future of health will require a more robust integration with environmental science research and policy, as drought is one of the most expensive natural events from a number of economic and public health vantage points (Cook et al., 2014). This must include, but certainly will not be limited to, the implications of population health associated with the most recent California drought and the receding water levels of the Salton Sea. The GeoHealth framework functions within other fields, including agriculture, economics, and the health and environmental sciences, among others. Therefore, the fate of the Aral Sea and the health of its surrounding human populations may be seen as a cautionary tale for Imperial County in Southern California and a guideline for future utilization of water resources for irrigation.

## Conflict of Interest

The authors declare no conflicts of interest relevant to this study.

## Data Availability

This manuscript is a literature review and does not present new data.

## References

[gh2174-bib-0001] Arballo, E. , Baza, M. , Mendoza, V. , Conde, M. , Cason, D. , Zavala, F. , & Gran, M. T. (2014). Imperial County comprehensive economic development strategy 2014–2015 annual update. El Centro, CA. Retrieved from http://www.co.imperial.ca.us/announcements%5CPDFs%5CCEDSpubliccomment.pdf

[gh2174-bib-0002] Aus Der Beek, T. , Voß, F. , & Flörke, M. (2011). Modelling the impact of global change on the hydrological system of the Aral Sea basin. Physics and Chemistry of the Earth, 36(13), 684–695. 10.1016/j.pce.2011.03.004

[gh2174-bib-0003] Barreau, T. , Conway, D. , Haught, K. , Jackson, R. , Kreutzer, R. , Lockman, A. , Minnick, S. , Roisman, R. , Rozell, D. , Smorodinsky, S. , Tafoya, D. , & Wilken, J. A. (2017). Physical, mental, and financial impacts from drought in two California counties, 2015. American Journal of Public Health, 107(5), 783–790. 10.2105/AJPH.2017.303695 28323464 PMC5388959

[gh2174-bib-0004] Bennion, P. , Hubbard, R. , O'hara, S. , Wiggs, G. , Wegerdt, J. , Lewis, S. , et al. (2007). The impact of airborne dust on respiratory health in children living in the Aral Sea region. International Journal of Epidemiology, 36(5), 1103–1110. 10.1093/ije/dym195 17911152

[gh2174-bib-0005] Bloudoff‐Indelicato, M. (2012, October). Climate change is bad news for California children with asthma. Scientific American.

[gh2174-bib-0006] Bureau, U. S. C . (2015). Quick facts: Imperial County, California. Retrieved June 11, 2017, from https://www.census.gov/quickfacts/table/PST045216/06025,00

[gh2174-bib-0007] Christian‐Smith, J. , Levy, M. C. , & Gleick, P. H. (2015). Maladaptation to drought: A case report from California, USA. Sustainability Science, 10(3), 491–501. 10.1007/s11625-014-0269-1

[gh2174-bib-0008] Cohen, M. J. (2014). Hazard's toll: The costs of inaction at the Salton Sea. Oakland, California. Retrieved from https://pacinst.org/wp-content/uploads/2014/09/PacInst_HazardsToll-1.pdf

[gh2174-bib-0009] Cohn, J. P. (2000). Saving the Salton Sea. Bioscience, 50(4), 295–301. Retrieved from. https://watermark.silverchair.com/api/watermark?token=AQECAHi208BE49Ooan9kkhW_Ercy7Dm3ZL_9Cf3qfKAc485ysgAAAfMwggHvBgkqhkiG9w0BBwagggHgMIIB3AIBADCCAdUGCSqGSIb3DQEHATAeBglghkgBZQMEAS4wEQQMZEY37CW8fSgt5RI3AgEQgIIBplB_vOpxrMW_BZ_aWeXr-WsRrU25KmJj0oqaNdqIlGjO, 10.1641/0006-3568(2000)050[0295:STSS]2.3.CO;2

[gh2174-bib-0010] Cook, B. I. , Smerdon, J. E. , Seager, R. , & Cook, E. R. (2014). Pan‐continental droughts in North America over the last millennium. Journal of Climate, 27(1), 383–397. 10.1175/JCLI-D-13-00100.1

[gh2174-bib-0011] Costello, A. , Abbas, M. , Allen, A. , Ball, S. , Bell, S. , Bellamy, R. , Friel, S. , Groce, N. , Johnson, A. , Kett, M. , Lee, M. , Levy, C. , Maslin, M. , McCoy, D. , McGuire, B. , Montgomery, H. , Napier, D. , Pagel, C. , Patel, J. , de Oliveira, J. A. P. , Redclift, N. , Rees, H. , Rogger, D. , Scott, J. , Stephenson, J. , Twigg, J. , Wolff, J. , & Patterson, C. (2009). Managing the health effects of climate change. Lancet and University College London Institute for Global Health Commission. The Lancet, 373(9676), 1693–1733. 10.1016/S0140-6736(09)60935-1 19447250

[gh2174-bib-0012] Crighton, E. J. , Elliott, S. J. , Upshur, R. , Van Der Meer, J. , & Small, I. (2003). The Aral Sea disaster and self‐rated health. Health & Place, 9(2), 73–82. Retrieved from https://ac.els-cdn.com/S1353829202000175/1-s2.0-S1353829202000175-main.pdf?_tid=ea2e6ccd-b16d-4c0e-af17-5b39e8b2ae4d%acdnat=1544726985_e5f29a23a24eacace8699a95c6c40116, 10.1016/S1353-8292(02)00017-5 12753790

[gh2174-bib-0013] D'Amato, G. , & Cecchi, L. (2008). Effects of climate change on environmental factors in respiratory allergic diseases. Clinical and Experimental Allergy, 38(8), 1264–1274. 10.1111/j.1365-2222.2008.03033.x 18537982

[gh2174-bib-0014] de Vlaming, V. , DiGiorgio, C. , Fong, S. , Deanovic, L. A. , de la Paz Carpio‐Obeso, M. , MIller, J. , et al. (2004). Irrigation runoff insecticide pollution of rivers in the Imperial Valley, California (USA). Environmental Pollution, 132(2), 213–229. 10.1016/j.envpol.2004.04.025 15312936

[gh2174-bib-0015] Frumkin, H. (2017). What Is Planetary Health and why Now. Boston: Planetary Health Alliance Annual Meeting.

[gh2174-bib-0016] Gauderman, J. W. , Avol, E. , Gilliland, F. , Vora, H. , Thomas, D. , Berhane, K. , et al. (2004). The effect of air pollution on lung development from 10 to 18 years of age. New England Journal of Medicine, 351(11), 1057–1067. 10.1056/NEJMoa040610 15356303

[gh2174-bib-0017] Gauderman, J. W. , Gilliland, G. F. , Vora, H. , Avol, E. , Stram, D. , McConnell, R. , et al. (2002). Association between air pollution and lung function growth in Southern California children: Results from a second cohort. American Journal of Respiratory and Critical Care Medicine, 166(1), 76–84. 10.1164/rccm.2111021 12091175

[gh2174-bib-0018] Glantz, M. , & Figueroa, R. (1997). Does the Aral Sea merit heritage status. Global Environmental Change, 7(4), 357–380.

[gh2174-bib-0019] Gomez, S. R. , Parker, R. A. , Dosman, J. A. , & McDuffie, H. H. (1992). Respiratory health effects of alkali dust in residents near desiccated Old Wives Lake. Archives of Environmental Health, 47(5), 364–369. 10.1080/00039896.1992.9938376 1444599

[gh2174-bib-0020] Goodyear, D. (2015, May). California runs dry. The New Yorker. Retrieved from http://www.newyorker.com/magazine/2015/05/04/the-dying-sea

[gh2174-bib-0021] Griffin, D. , & Anchukaitis, K. J. (2014). How unusual is the 2012–2014 California drought? Geophysical Research Letters, 41, 9017–9023. 10.1002/2014GL062433.1

[gh2174-bib-0022] Grigoryev, A. A. (1952). Soviet plans for irrigation and power: A geographical assessment. Source: The Geographical Journal, 118). Retrieved from. https://www.jstor.org/stable/pdf/1791946.pdf?refreqid=excelsior%3A80131dc04cb30f44c7f089f7744a5125

[gh2174-bib-0023] Hefflin, B. J. , Jalaludin, B. , McClure, E. , Cobb, N. , Johnson, C. A. , Jecha, L. , & Etzel, R. A. (1991). Surveillance for dust storms and respiratory diseases in Washington state, 1991. Archives of Environmental Health, 49(3), 170–174. 10.1080/00039896.1994.9940378 8185387

[gh2174-bib-0024] Indoitu, R. , Kozhoridze, G. , Batyrbaeva, M. , Vitkovskaya, I. , Orlovsky, N. , Blumberg, D. , & Orlovsky, L. (2015). Dust emission and environmental changes in the dried bottom of the Aral Sea. Aeolian Research, 17, 101–115. 10.1016/j.aeolia.2015.02.004

[gh2174-bib-0025] Johnston, J. E. , Razafy, M. , Lugo, H. , Olmedo, L. , & Farzan, S. F. (2019). The disappearing Salton Sea: A critical reflection on the emerging environmental threat of disappearing saline lakes and potential impacts on children's health. Science of the Total Environment, 663, 804–817. 10.1016/j.scitotenv.2019.01.365 30738261 PMC7232737

[gh2174-bib-0026] Kaiser, J. (1999). Battle over a dying sea. Science, 284(5411), 28–30. 10.1126/science.284.5411.28

[gh2174-bib-0027] Kaneko, K. , Chiba, M. , Hashizume, M. , Kunii, O. , Sasaki, S. , Shimoda, T. , Yamashiro, Y. , Caypil, W. , & Dauletbaev, D. (2003). Renal tubular dysfunction in children living in the Aral Sea region. Archives of Disease in Childhood, 88(11), 966–968. 10.1136/adc.88.11.966 14612357 PMC1719339

[gh2174-bib-0028] Kelley, C. P. , Mohtadi, S. , Cane, M. A. , Seager, R. , & Kushnir, Y. (2015). Climate change in the Fertile Crescent and implications of the recent Syrian drought. Proceedings of the National Academy of Sciences, 112(11), 3241–3246. 10.1073/pnas.1421533112 PMC4371967 25733898

[gh2174-bib-0029] Ketcham, C. (2012). Razing Arizona: Will drought destroy the Southwest? Harper's Magazine.

[gh2174-bib-0030] King, J. , Etyemezian, V. , Sweeney, M. , Buck, B. J. , & Nikolich, G. (2011). Dust emission variability at the Salton Sea, California, USA. Aeolian Research, 3(1), 67–79. 10.1016/j.aeolia.2011.03.005

[gh2174-bib-0031] Kunii, O. , Hashizume, M. , Chiba, M. , Sasaki, S. , & Shimoda, T. (2010). Respiratory symptoms and pulmonary function among school‐age children in the Aral Sea region. Archives of Environmental Health, 58(11), 676–682. 10.3200/AEOH.58.11.676-682 15702891

[gh2174-bib-0032] Laylander, D. (1995). The chronology of Lake Cahuilla's final stand. Proceedings of the Society for California Archaeology, 8, 69–78. Retrieved from. https://scahome.org/publications/proceedings/Proceedings.08Laylander.pdf

[gh2174-bib-0033] Lee, S. O. , & Jung, Y. (2018). Efficiency of water use and its implications for a water‐food nexus in the Aral Sea basin. Agricultural Water Management, 207, 80–90. 10.1016/j.agwat.2018.05.014

[gh2174-bib-0034] Myers, S. S. (2017). Planetary health: Protecting human health on a rapidly changing planet. Lecture 2860 Www.Thelancet.Com, 390. 10.1016/S0140-6736(17)32846-5 29146123

[gh2174-bib-0035] Observatory, N. E. (2007). Dust storm over the South Aral Sea. Retrieved from https://earthobservatory.nasa.gov/images/18359/dust-storm-over-the-south-aral-sea

[gh2174-bib-0036] Observatory, N. E. (2012). Shrinking Aral Sea. Retrieved from https://svs.gsfc.nasa.gov/vis/a010000/a010800/a010862/index.html

[gh2174-bib-0037] Observatory, N. E. (2015). Shrinking shoreline of the Salton Sea. Retrieved from https://earthobservatory.nasa.gov/images/86746/shrinking-shoreline-of-the-salton-sea

[gh2174-bib-0038] O'Connor, T. , Hsia‐Kiung, K. , Koehler, L. , Holmes‐Gen, B. , Barrett, W. , Chan, M. , & Law, K. (2014). Driving California forward: Public health and societal economic benefits of California's AB 32 transportation fuel policies.

[gh2174-bib-0039] O'Hara, S. L. (2000). Central Asia's water resources: Contemporary and future management issues. International Journal of Water Resources Development, 16(3), 423–441. 10.1080/713672501

[gh2174-bib-0040] O'Hara, S. L. , Wiggs, G. F. S. , Mamedov, B. , Davidson, G. , & Hubbard, R. B. (2000). Exposure to airborne dust contaminated with pesticide in the Aral Sea region. Lancet, 355(9204), 627–628. 10.1016/S0140-6736(99)04753-4 10696990

[gh2174-bib-0041] Orlando, J. L. , Smalling, K. L. , & Kuivila, K. M. (2006). Pesticides in water and suspended sediment of the Alamo and New Rivers *,* Imperial Valley/Salton Sea Basin. Retrieved from https://pubs.usgs.gov/ds/365/pdf/ds365.pdf

[gh2174-bib-0042] Program, U. N. E. (1992). The Aral Sea: Diagnostic Study for the Development of an Action Plan for the Conservation of the Aral Sea. Nairobi: United Nations Environmental Programme.

[gh2174-bib-0043] Sarfaty, M. , Bloodhart, B. , Ewart, G. , Thurston, G. D. , Balmes, J. R. , Guidotti, T. L. , & Maibach, E. W. (2015). American Thoracic Society member survey on climate change and health. Annals of the American Thoracic Society, 12(2), 274–278. 10.1513/AnnalsATS.201410-460BC 25535822 PMC5466202

[gh2174-bib-0044] Shukla Mcdermid, S. , & Winter, J. (2017). Anthropogenic forcings on the climate of the Aral Sea: A regional modeling perspective. 20, 48–60. 10.1016/j.ancene.2017.03.003

[gh2174-bib-0045] Smith, L. T. , Aragão, L. E. O. C. , Sabel, C. E. , & Nakaya, T. (2014). Drought impacts on children's respiratory health in the Brazilian Amazon. Scientific Reports, 4(1), 3726 10.1038/srep03726 24430803 PMC3893650

[gh2174-bib-0046] State of California Department of Water Resources (2017). Governor's drought declaration. Retrieved June 13, 2017, from http://www.water.ca.gov/waterconditions/declaration.cfm

[gh2174-bib-0047] The Economist (2014). The drying of the West: Drought is forcing Westerners to consider wasting less water. *The Economist*, (February 22). Retrieved from http://www.economist.com/news/united-states/21596955-drought-forcing-westerners-consider-wasting-less-water-%0Adrying-west%0A

[gh2174-bib-0048] The Economist (2015). Cut and dried: Civilians will bear the brunt of new water restrictions, though it is teh farms that use the most. *The Economist*, (August 8). Retrieved from http://www.economist.com/news/united-states/21647812-governor-cut-and-dried

[gh2174-bib-0049] Whish‐Wilson, P. (2002). The Aral Sea environmental health crisis. Journal of Rural and Remote Environmental Health, 1(2), 29–34.

[gh2174-bib-0050] White, K. D. (2013). Nature‐society linkages in the Aral Sea region. Journal of Eurasian Studies, 4(1), 18–33. 10.1016/j.euras.2012.10.003

[gh2174-bib-0051] Wiggs, G. F. S. , O'Hara, S. L. , Wegerdt, J. , van der Meer, J. , Small, I. , & Hubbard, R. (2003). The dynamics and characteristics of aeolian dust in dryland central Asia: Possible impacts on human exposure and respiratory health in the Aral Sea basin. Geographical Journal, 169(2), 142–157. 10.1111/1475-4959.04976

[gh2174-bib-0052] Xu, E. G. , Bui, C. , Lamerdin, C. , & Schlenk, D. (2016). Spatial and temporal assessment of environmental contaminants in water, sediments and fish of the Salton Sea and its two primary tributaries, California, USA, from 2002 to 2012. Science of the Total Environment, 559, 130–140. 10.1016/j.scitotenv.2016.03.144 27058132

[gh2174-bib-0053] Young, A. (2016, June). California is now world's 6th‐largest economy. Sacramento Business Journal.

